# Supplementary material for: Small extracellular vesicles derived from microRNA-22-3p-overexpressing mesenchymal stem cells protect retinal ganglion cells by regulating MAPK pathway
Source: Commun Biol. 2024 Jul 3;7:807. doi: 10.1038/s42003-024-06511-z (PMC11222527; doi:10.1038/s42003-024-06511-z)

Supplementary Table 1 Primers of detected miRNAs and mRNAs

	Forward	Reverse
U6	5’-CTCGCTTCGGCAGCACATATACT-3’	5’-ACGCTTCACGAATTTGCGTGTC-3’
miR22	5’-ACACTCCAGCTGGGAAGCTGCCAGTTGAAG-3’	5’-TGGTGTCGTGGAGTCG-3’
GAPGH	5’-TGTGTCCGTCGTGGATCTGA-3’	5’-CCTGCTTCACCACCTTCTTGA-3’
Bax	5’-TGCTACAGGGTTTCATCCA-3’	5’-AAGTAGAAGAGGGCAACCAC-3’
caspase-3	5’-ATGGAGAACAACAAAACCTCAGT-3’	5’-TTGCTCCCATGTATGGTCTTTAC-3’
Max	5’-AGCGAGGTTTCAATCTGCGG-3’	5’-ACGTTTTCGTTCCAGTGCATTAT-3’
Arrb1	5’-AAGGGACACGAGTGTTCAAGA-3’	5’-CCCGCTTTCCCAGGTAGAC-3’
Csf1r	5’-TGTCATCGAGCCTAGTGGC-3’	5’-CGGGAGATTCAGGGTCCAAG-3’
Map3k12	5’-ATGGCCTGCCTCCATGAAAC-3’	5’-GGGCGTCAGGTCTTTCTCG-3’
Akt3	5’-TGGGTTCAGAAGAGGGGAGAA-3’	5’-AGGGGATAAGGTAAGTCCACATC-3’
Tgfbr1	5’-TCTGCATTGCACTTATGCTGA-3’	5’-AAAGGGCGATCTAGTGATGGA-3’

Supplementary Figure 1. Gating strategy for the analysis of apoptosis using Annexin V and PI staining in RGC-5.

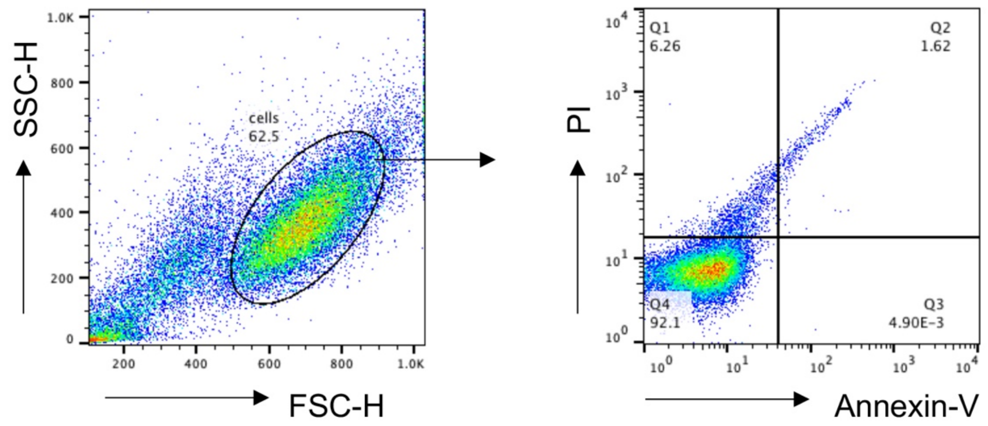

Supplementary Figure 2. Original western blots

Raw Western Blot scans in Figure 1d

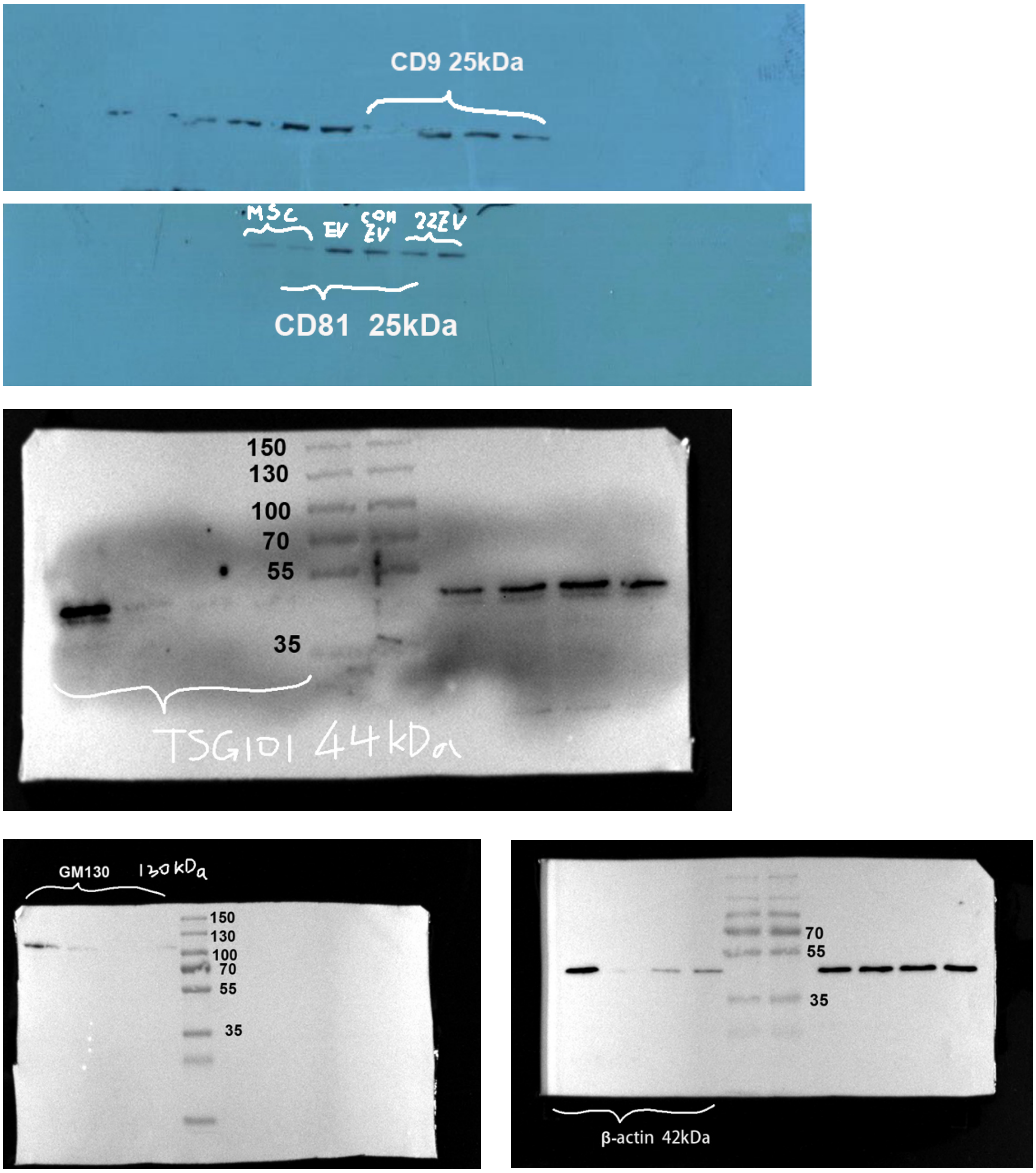

Raw Western Blot scans in Figure 3c

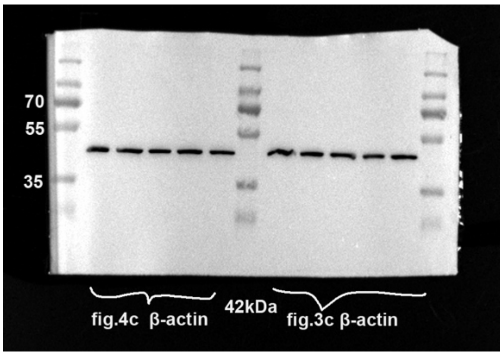

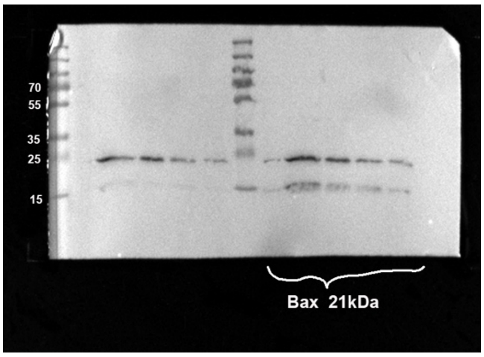

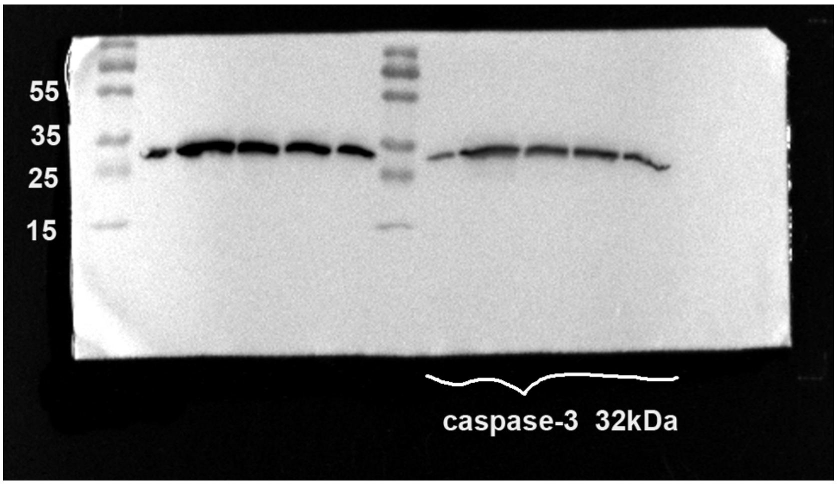

Raw Western Blot scans in Figure 4c

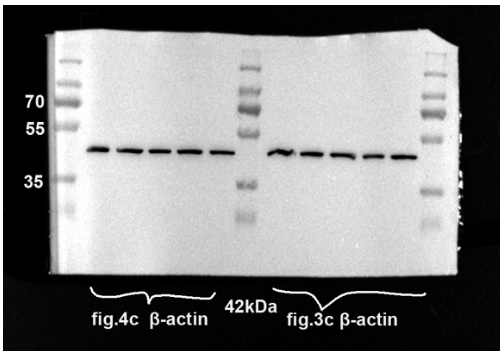

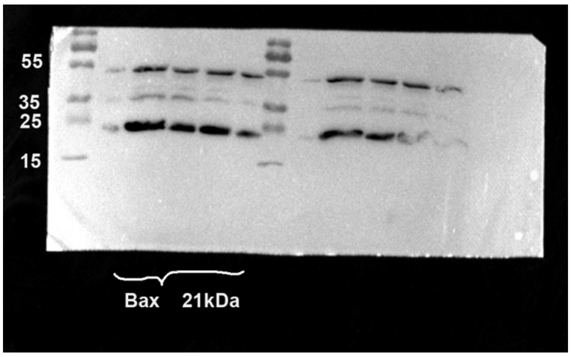

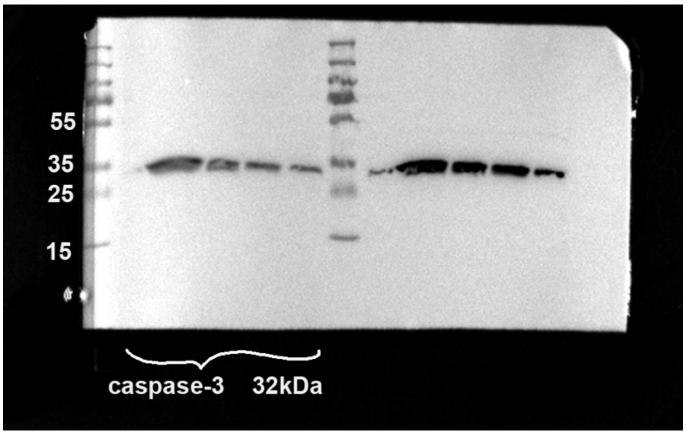

Raw Western Blot scans in Figure 4e

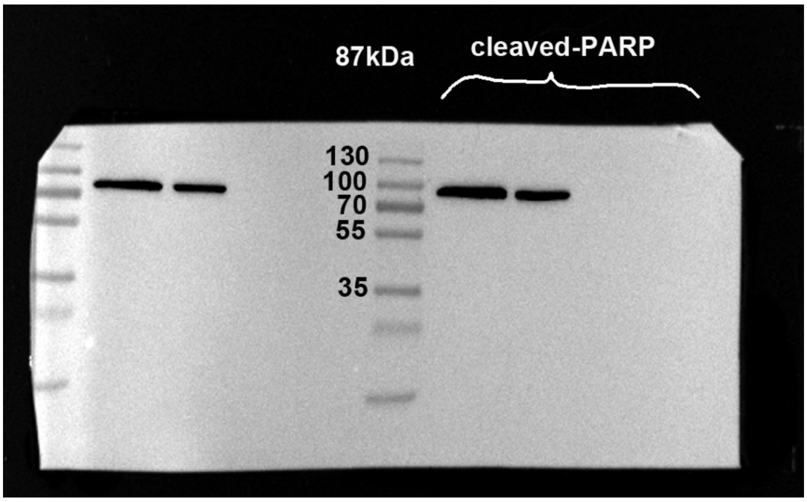

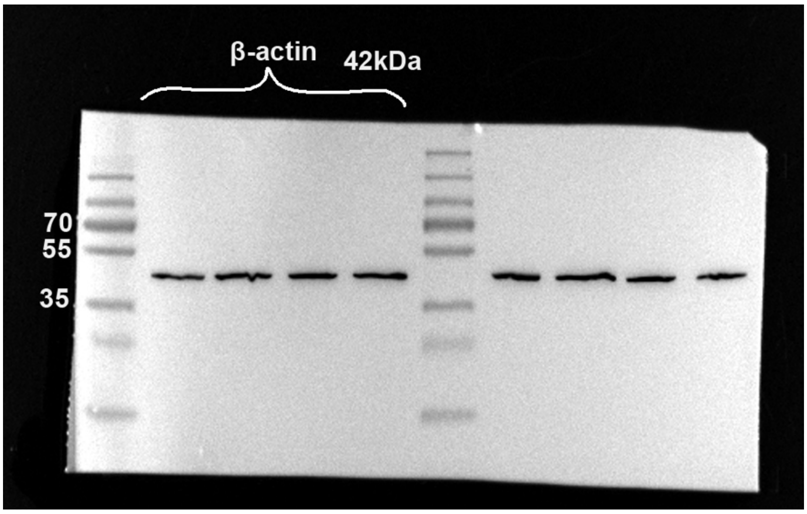

Raw Western Blot scans in Figure 6d

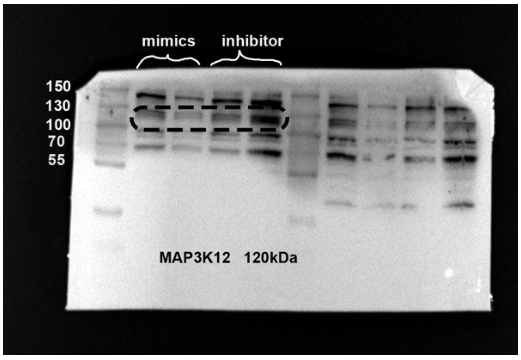

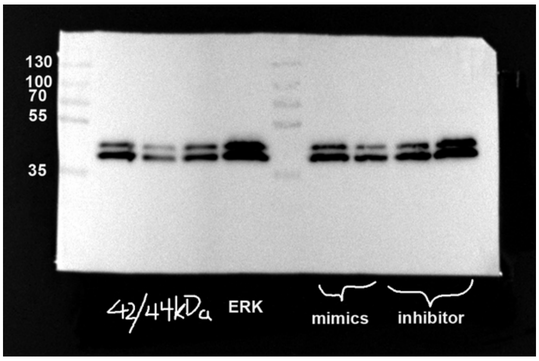

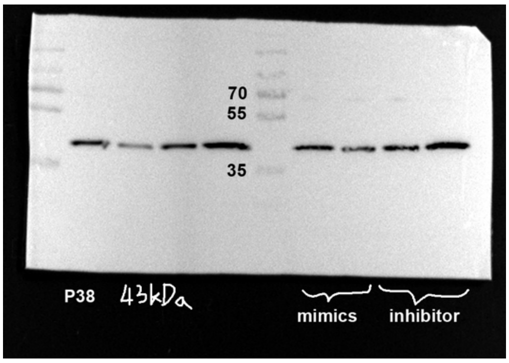

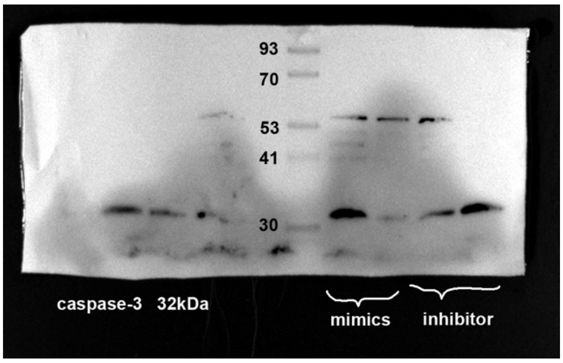

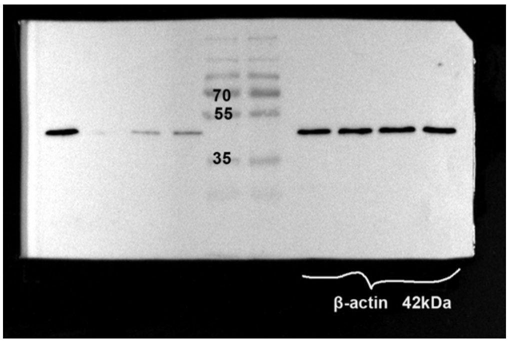

Supplement: Supplementary file 2 — Supplementary information [file 42003_2024_6511_MOESM2_ESM.pdf]
