# Supplementary material for: Small extracellular vesicles derived from microRNA-22-3p-overexpressing mesenchymal stem cells protect retinal ganglion cells by regulating MAPK pathway
Source: Commun Biol. 2024 Jul 3;7:807. doi: 10.1038/s42003-024-06511-z (PMC11222527; doi:10.1038/s42003-024-06511-z)
Supplement: Supplementary file 5 — reporting summary [file 42003_2024_6511_MOESM5_ESM.pdf]

nature portfolio

Corresponding author(s): Xiaomin Zhang, Xiaoli Xing

Last updated by author(s): Jun 12, 2024

Reporting Summary

Nature Portfolio wishes to improve the reproducibility of the work that we publish. This form provides structure for consistency and transparency in reporting. For further information on Nature Portfolio policies, see our Editorial Policies and the Editorial Policy Checklist.

Statistics

For all statistical analyses, confirm that the following items are present in the figure legend, table legend, main text, or Methods section.

n/a	Confirmed	
☐	☒	The exact sample size (*n*) for each experimental group/condition, given as a discrete number and unit of measurement
☐	☒	A statement on whether measurements were taken from distinct samples or whether the same sample was measured repeatedly
☐	☒	The statistical test(s) used AND whether they are one- or two-sided<br>*Only common tests should be described solely by name; describe more complex techniques in the Methods section.*
☒	☐	A description of all covariates tested
☐	☒	A description of any assumptions or corrections, such as tests of normality and adjustment for multiple comparisons
☐	☒	A full description of the statistical parameters including central tendency (e.g. means) or other basic estimates (e.g. regression coefficient) AND variation (e.g. standard deviation) or associated estimates of uncertainty (e.g. confidence intervals)
☒	☐	For null hypothesis testing, the test statistic (e.g. *F*, *t*, *r*) with confidence intervals, effect sizes, degrees of freedom and *P* value noted<br>*Give P values as exact values whenever suitable.*
☒	☐	For Bayesian analysis, information on the choice of priors and Markov chain Monte Carlo settings
☒	☐	For hierarchical and complex designs, identification of the appropriate level for tests and full reporting of outcomes
☒	☐	Estimates of effect sizes (e.g. Cohen's *d*, Pearson's *r*), indicating how they were calculated

Our web collection on statistics for biologists contains articles on many of the points above.

Software and code

Policy information about availability of computer code

Data collection	No software was used in the study to collect data.
Data analysis	All data were analyzed by using GraphPad Prism10 (GraphPad Software, Inc., La Jolla, CA92037, USA).

For manuscripts utilizing custom algorithms or software that are central to the research but not yet described in published literature, software must be made available to editors and reviewers. We strongly encourage code deposition in a community repository (e.g. GitHub). See the Nature Portfolio guidelines for submitting code & software for further information.

Data

Policy information about availability of data

All manuscripts must include a data availability statement. This statement should provide the following information, where applicable:

- Accession codes, unique identifiers, or web links for publicly available datasets
- A description of any restrictions on data availability
- For clinical datasets or third party data, please ensure that the statement adheres to our policy

All data supporting the findings of this study are available within the paper or in the Supplementary Information file, including the source data as original western blots in Supplementary Fig. 2. All data are available from the corresponding author upon a reasonable request.

Research involving human participants, their data, or biological material

Policy information about studies with human participants or human data. See also policy information about sex, gender (identity/presentation), and sexual orientation and race, ethnicity and racism.

Reporting on sex and gender	The research doesn't involve human participants, their data or biological material.
Reporting on race, ethnicity, or other socially relevant groupings	The research doesn't involve human participants, their data or biological material.
Population characteristics	The research doesn't involve human participants, their data or biological material.
Recruitment	The research doesn't involve human participants, their data or biological material.
Ethics oversight	The research doesn't involve human participants, their data or biological material.

Note that full information on the approval of the study protocol must also be provided in the manuscript.

Field-specific reporting

Please select the one below that is the best fit for your research. If you are not sure, read the appropriate sections before making your selection.

☒ Life sciences ☐ Behavioural & social sciences ☐ Ecological, evolutionary & environmental sciences

For a reference copy of the document with all sections, see nature.com/documents/nr-reporting-summary-flat.pdf

Life sciences study design

All studies must disclose on these points even when the disclosure is negative.

Sample size	Sample sizes were chosen based on our preliminary experiments to assure adequate statistical power. The exact sample size of each experiment is described in the figure legend.
Data exclusions	No data was excluded.
Replication	All experiments were repeated at least 3 times.
Randomization	Mice were randomly divided into five groups (the control group, the PBS group, the MSC-sEVs group, the con-sEVs group, and the miR22-sEVs group) using a random number table.
Blinding	Investigators were not blinded to allocation during experiments and assessment.

Reporting for specific materials, systems and methods

We require information from authors about some types of materials, experimental systems and methods used in many studies. Here, indicate whether each material, system or method listed is relevant to your study. If you are not sure if a list item applies to your research, read the appropriate section before selecting a response.

Materials & experimental systems

n/a	Involved in the study	
☐	☒	Antibodies
☒	☐	Eukaryotic cell lines
☒	☐	Palaeontology and archaeology
☐	☒	Animals and other organisms
☒	☐	Clinical data
☒	☐	Dual use research of concern
☒	☐	Plants

Methods

n/a	Involved in the study	
☒	☐	ChIP-seq
☐	☒	Flow cytometry
☒	☐	MRI-based neuroimaging

Antibodies

Antibodies used	The supplier names and catalog number of all antibodies were provided in the Methods part where they appeared.

Validation	All antibodies used are commercially available and have been validated for the application used by the manufacturer. References can be found on the manufacturers' website.

Animals and other research organisms

Policy information about studies involving animals; ARRIVE guidelines recommended for reporting animal research, and Sex and Gender in Research

Laboratory animals	Female C57BL/6 mice (6-8 weeks of age) were purchased from Vital River Experimental Animal Center (Beijing, China)
Wild animals	The study didn't involve wild animals.
Reporting on sex	Female mice were used in the study according to previous literatures.
Field-collected samples	The study didn't involve samples collected from field.
Ethics oversight	All experimental procedures were in accordance with the statement for the use of animals in ophthalmic and vision research of the Association for Research in Vision and Ophthalmology (ARVO), and approved by the Laboratory Animal Care and Use Committee of Tianjin Medical University Eye Hospital (No. TJYY20201221035).

Note that full information on the approval of the study protocol must also be provided in the manuscript.

Plants

Seed stocks	not involved
Novel plant genotypes	not involved
Authentication	not involved

Flow Cytometry

Plots

Confirm that:

☒ The axis labels state the marker and fluorochrome used (e.g. CD4-FITC).

☒ The axis scales are clearly visible. Include numbers along axes only for bottom left plot of group (a 'group' is an analysis of identical markers).

☒ All plots are contour plots with outliers or pseudocolor plots.

☒ A numerical value for number of cells or percentage (with statistics) is provided.

Methodology

Sample preparation	Cells were diluted with the binding buffer into a concentration of 106 cells/mL.
Instrument	After cells were resuspended in PBS, flow cytometric analysis was performed with a BD FACSCalibur.
Software	Data were analyzed using flowjo_v10 software (FlowJo, LLC, USA).
Cell population abundance	Purity of the sorted cells was determined for each group of sorted cells using the same sorted instrument. The purity was over 95%.
Gating strategy	Gating strategy was shown in Supplementary Figure 1.

☒ Tick this box to confirm that a figure exemplifying the gating strategy is provided in the Supplementary Information.
